# Romantic Relationship Dissolution, Microbiota, and Fibers

**DOI:** 10.3389/fnut.2021.655038

**Published:** 2021-04-15

**Authors:** Jie-Yu Chuang

**Affiliations:** ^1^Department of Psychiatry, Cardinal Tien Hospital, New Taipei City, Taiwan; ^2^School of Medicine, College of Medicine, Fu Jen Catholic University, New Taipei City, Taiwan

**Keywords:** romantic relationship, microbiota, microbiome, love, gut-microbiota-brain axis, fiber, romantic relationship dissolution

## Abstract

Microbiota inhabit nearly every part of our body with the gut microbiota representing the greatest density and absolute abundance. The gut-microbiota-brain axis facilitates bidirectional communication between gut microbiota and the brain. For instance, romantic relationship not only brings joy, it is also associated with increased gut microbiota diversity and health benefits whereas reduced microbiota diversity is related to obesity, cardiac disease, type 2 diabetes, and inflammatory disorders. Research has shown that dietary fibers may increase microbiota diversity and exert antidepressant effect. Among a plethora of life stressors, romantic relationship dissolution is a relatively common and painful experience that people encounter from time to time. Depressed mood, social isolation and poor intake are all associated with romantic relationship dissolution. In this article, it is hypothesized that romantic relationship dissolution is accompanied by decreased gut microbiota diversity which could be corrected with the ingestion of dietary fibers with an additional antidepressant benefit.

## Gut-Microbiota-Brain Axis, Stress and Diet

Microbiota inhabit nearly every part of our body with the gut microbiota representing the greatest density and absolute abundance (1). It remains elusive what definitively constitutes an optimal gut microbial profile other than one exhibiting both stability and diversity (2). Microbiota diversity may promote stability and resilience of gut microbiota and is associated with social interaction, infection resistance, improved immunity and reduced inflammation (3). Reduced microbiota diversity is related to obesity, cardiac disease, type 2 diabetes, and inflammatory disorders (4).

The gut-microbiota-brain axis denotes the bidirectional interaction between gut microbiota and brain. Microbiota can produce neurotransmitters (GABA, oxytocin, noradrenaline, dopamine, serotonin, etc.), amino acids (tyramine, tryptophan, etc.) and metabolites (short-chain fatty acids, etc.) which travel through portal circulation to influence immune system, enteric nervous system, gut barrier integrity and the central nervous system (presumably via vagus nerve) (1). Conversely, in response to stress, the hypothalamic-pituitary-adrenal axis can produce cortisol affecting the intestinal barrier integrity, changing the gut environment, and finally altering the gut microbiota composition (1) resulting in a reduction of microbiota diversity (5). For instance, fecal lactic acid bacterial levels are found to be significantly reduced when undergraduate students experience high academic stress (6). Similarly, marital distress has been associated with increased gut permeability (7), possibly changing gut microbiota composition. Moreover, stress might prompt unhealthy food choices, alter metabolic responses to food, and adjust microbiota constitution (8).

Environmental factors and health behaviors might explain more microbiota variability than do genetic factors (9). Among these environmental factors, diet has emerged as one of the major predictors of gut microbiota composition (8). To confer a health benefit, prebiotic (such as fibers) is dietary supplement which is selectively utilized by microbiota whereas probiotic (such as *Lactobacillus*) is live microorganism ingested (2). Recently, there has been a surge in studies exploring the beneficial effects of prebiotics and probiotics on mental health (2). Nevertheless, results from these studies are only slowly being translated from animals to humans (10). Unstable results are reported in a recent meta-analysis exploring the efficacy of probiotics on stress reaction with the authors urging more large-scale and well-designed clinical trials in the future (11). Technical difficulties with probiotics include: ensuring the survival of probiotics to reach and colonize the intestines, and selection of appropriate bacterial strains (2). Intervention with prebiotics can globally improve gut microbiota status in contrast to the use of specific probiotics (2). In sum, an optimal diet therapy mitigating the detrimental effect of stress on gut-microbiota-brain axis remains undetermined.

## Romantic Relationship Dissolution

In 1967, Holmes and Rahe developed the Social Readjustment Rating Scale to identify major life stressors. Among the 43 life stressors, romantic relationship dissolution (such as death of spouse or divorce) has been found to be the most traumatic one (12). Nearly 85% of people experience at least one romantic relationship dissolution in their lifetime (13). This universal experience might lead to grief and depression (14). Romantic relationship dissolution has been referred to as “heartbreak” or “post-relationship grief” (15). Indeed, some even commit suicide following the termination of romantic relationships (16). Romantic relationship dissolution might be accompanied by anxiety, fear, anger, panic, worry, sadness, emotional numbness, loss of purpose, poor concentration, poor memory, poor function, and various somatic symptoms such as loss of appetite and even impaired immunity (15). Copious studies have shown compromised immune function in divorced compared to married adults, such as higher antibody titers to Epstein-Barr virus and lower percentage of natural killer cell activity (17, 18). Furthermore, aberrant brain activations in the cerebellum, insula, pre-frontal cortex, anterior temporal cortex, and anterior cingulate after romantic relationship dissolution have been documented (19).

To sum up, romantic relationship dissolution is a universal and excruciating life stressor impacting both mental and physical health. With the emergence of COVID-19 pandemic, a deterioration of romantic relationship has been found by large-scale, cross-sectional online questionnaire studies in both US (20) and China (21). Presumably, many people might experience romantic relationship dissolution, and some people might need professional mental help. Nevertheless, due to quarantine and home confinement, opportunities to deliver professional clinical support via direct encounters are greatly curtailed in this crisis (22). Accordingly, this article introduces a self-help and easily-implemented hypothetical treatment for romantic relationship dissolution, as described in the following sections.

## Romantic Relationship Dissolution and Microbiota

Couples demonstrate more similar gut microbiota than siblings (4). Therefore, it has been postulated that romantic relationship holds a strong influence on gut microbiota, possibly more powerful than shared genetics and early life environments among siblings (4). The mechanism linking romantic relationship to microbiota remains elusive with some speculations. Firstly, couples might share their microbiota via kissing. The microbiota on the dorsal surface of the tongue has been found to be more similar among partners (Morisita-Horn index = 0.37) than unrelated individuals (Morisita-Horn index = 0.55) (23). Indeed, salivary microbiota is more similar with higher frequency of kissing between couples (23). Secondly, another study shows that transfer of microbiota between couples might occur through shared environment and direct skin contact as cohabiting couples demonstrate similar skin microbiota, especially on their feet, likely reflecting collection of dust (24). Thirdly, behavioral concordance might result in the similarity between partner, such as diet, exercise, sleep, smoking, and alcohol consumption (25). Indeed, diet has a major impact on gut microbiota (26), and dietary patterns play a significant role in the shared gut microbiota in couples (27). Consequently, the above-mentioned evidence might suggest the considerable impact of romantic relationship on gut microbiota.

One study found a significant correlation between larger social network size and increased microbiota diversity (3). It has been speculated that social interaction allows microbiota transmission between subjects and contributes to increased microbiota diversity which counterbalances sociability-related enhanced exposure to infectious agents (5). Indeed, cohabiting spouses, especially those with close relationships, are shown to have more microbiota diversity than those living alone (4). Opposingly, stress has been associated with a reduction of microbiota diversity (5), especially interpersonal stress (conflict, romantic relationship dissolution, etc.) might arise during social interaction and result in a reduction of microbiota diversity (5). Furthermore, it has been demonstrated that depression is also associated with decreased gut microbiota diversity (28). Given that romantic relationship dissolution is frequently associated with solitary living, stressful feeling, and depressive tendency (29), it is speculated that romantic relationship dissolution might be related to decreased microbiota diversity.

In short, studies have suggested that romantic relationship might significantly influence microbiota and is associated with increased microbiota diversity (presumably due to shared diet or microbiota transfer between couples, such as kissing and touching) than singleton. Despite lack of studies directly exploring the interaction between romantic relationship dissolution and microbiota, it is hypothesized that romantic relationship dissolution might be related to decreased microbiota diversity.

## Romantic Relationship Dissolution, Microbiota and Fibers: The Hypothesis

Diet has a major impact on gut microbiota diversity (26). Dietary fibers are considered as prebiotic, and is associated with increased microbiota diversity (26). Dietary fibers include non-starch polysaccharides, resistant oligosaccharides, lignin, and resistant starch (30). Dietary fiber is defined as edible carbohydrate polymer with three or more monomeric units that are resistant to the endogenous digestive enzymes and neither hydrolyzed nor absorbed in the small intestine (26). Subsequently, gut bacteria can degrade dietary fibers and provide a plethora of simple oligomers serving as an energy source for fermentative microbiota and promote the diversification of gut microbiota (31). Short-chain fatty acids (SCFAs) can then be fermented from fibers by gut microbiota. SCFAs stimulate mucus production, promote generation of colonic regulatory T cells, increase colonic mineral absorption (26), induce gluconeogenesis, and serve as an energy source for colonocyte (32).

Dietary fiber potentially reduces inflammation (via modifying pH and the gut permeability) resulting in alteration of the neurotransmitter concentration (30). Indeed, dietary fiber intake has been found to inversely associated with depression (30, 33, 34). Several potential antidepressant mechanisms of dietary fibers have been speculated: (1) SCFAs produced by gut microbiota fermentation of dietary fibers can inhibit histone deacetylases and increase antidepressant brain-derived neurotrophic factor (BDNF) (2) SCFAs might activate G-protein-coupled receptor and increase antidepressant norepinephrine (3) Fiber intake might potentiate gut microbiota to produce more antidepressant tryptophan, serotonin, and GABG (4) SCFAs can decrease gut permeability with the subsequent decreased serum level of bacteria-produced lipopolysaccharides leading to reduced inflammation whereas inflammation is associated with depression (5) Bicarbonate is released during the production of SCFAs leading to a reduction in intestinal pH and inhibiting lipopolysaccharides-producing bacteria (30).

In sum, it is hypothesized that romantic relationship dissolution is associated with significantly decreased gut microbiota diversity which might be corrected via dietary fiber ingestion with an additional antidepressant effect.

## Discussion

Romantic relationship dissolution is seldom addressed in scientific literature in spite of its enormous impact to emotional and physical health. This article presents a preliminary hypothesis linking romantic relationship dissolution with gut microbiota and dietary fibers.

To test this hypothesis, several issues should be considered. Firstly, influences of fibers on the gut microbiota has a significant interpersonal variation (35). Some might have problems tolerating high doses of fibers (flatulence, bloating, stomachaches, etc.) (26). Consequently, instead of a one-fits-all high-fiber diet, future studies might focus on designing a personalized high-fiber diets for people after romantic relationship dissolution (current dietary recommendation of dietary fibers is around 25–30 g/day (32), higher amount is suggested for romantic relationship dissolution). Secondly, instead of stress-provoking, romantic relationship dissolution can sometimes be stress-relieving and not-applicable for this hypothesis. Thirdly, despite potential biases in participant recruitment and study design, sex differences in emotional responsiveness (36) and resilience (37) to romantic relationship dissolution are suggested. A significant sex difference in depression prevalence is also well-acknowledged (38). Moreover, sex differences have also been found in microbiota diversity (3), degree of microbiota similarity with family members/spouse (39), and effect of fiber ingestion on microbiota (40). Therefore, sex difference in the interaction between romantic relationship dissolution, microbiota, and impact of fibers warrant careful exploration in future studies.

Stress has a great impact on microbiota. Although romantic relationship dissolution stands out as one of the most traumatic life stressors (12), other stressors might also be related to change of dietary habits, lack of physical contacts with others and depressed mood. Consequently, these stressors might influence microbiota to the similar extent as romantic relationship dissolution. For instance, a significant change in microbial environment is expected when one moves out of one's parents' house to live alone in a new city. Similar extent of microbiota alteration can also be found when one is under quarantine in a foreign country and devoid of physical contacts with others. In these situations, fiber supplementation might be able to counteract the effect of stress on gut microbiota diversity. Future studies are warranted to explore the interaction between various life stressors, microbiota, and fibers.

## Data Availability Statement

The original contributions presented in the study are included in the article/supplementary material, further inquiries can be directed to the corresponding author/s.

## Author Contributions

J-YC developed the hypothesis and wrote the manuscript.

## Conflict of Interest

The author declares that the research was conducted in the absence of any commercial or financial relationships that could be construed as a potential conflict of interest.
